# Transforming health through the metaverse

**DOI:** 10.1177/01410768221144763

**Published:** 2022-12-08

**Authors:** Connor S Qiu, Azeem Majeed, Sadhia Khan, Mando Watson

**Affiliations:** 1 School of Public Health, Imperial College London, London SW7 2AZ, UK; 2 Department of Paediatrics, Imperial College Healthcare NHS Trust, London W2 1NY, UK

A real change is on the horizon. In October 2021, Facebook announced that it would rebrand itself as ‘Meta’, and this generated high levels of public interest in the metaverse for the first time. Definitions for the metaverse vary and there is still much uncertainty in its eventual future manifestation. It is perhaps best defined as a fully immersive parallel digital reality where users will be able to interact at a scale previously unimagined.^1^ The advent of the metaverse could have transformational impact on every aspect of human life, from our social interactions to what we ascribe real value to. Just as the Internet has completely transformed health, the metaverse will redefine virtual and physical possibilities in health.^2^ This will have major implications for our health and for healthcare delivery. The coming of age of the metaverse is in due largely to the maturation of technological advances in artificial intelligence and devices that enable the delivery of mixed, augmented and virtual reality, along with cryptography, the catalyst behind web3, and increased computing power.^1^

The concept of the metaverse is not new. One of the first incarnations of a virtual world that attempted to mimic many of the activities in the real world is the video game *Second Life*, which was first launched in 2003. In this game, players from all over the world, called *residents*, would style and create a representation of themselves, known as *avatars*. There was also a native currency, the Linden dollar, which could be exchanged for real money.

This video game may seem far detached from the desire of health systems to improve a population’s physical and mental health, but Second Life has been a platform where diverse health initiatives have been trialled, including for health education, advocacy and training.^3^ These include the Cystic Fibrosis University, which is an example of a patient health education initiative.^3^ A Diabetes UK headquarters in the virtual world and patient support networks also exist; for example, for families of disabled children. Membership of such a virtual world could increase the psychological resilience of people against world-changing events, illustrated during the advent of the COVID-19 pandemic, and its uncertain implications for individual health and wellbeing.^4^ As virtual world behaviours can translate into real-world behaviours and actions, and as these experiences become increasingly immersive, the Proteus effect, where users take on the attributes of their virtual incarnations, could be a progressively important and powerful way of changing the global health dialogue and health attitudes in the future.^5^

There have already been numerous attempts to use more immersive, dynamic and mobile computing experiences to revitalise healthcare services and delivery. Accelerated by the ongoing COVID-19 pandemic, telehealth has seen considerable growth and illustrates some of the concerns and barriers of technological adoption. Remote healthcare delivery, and the monitoring of medical conditions such as hypertension and depression, levels the playing field in terms of quality of delivery through expertise sharing and accessibility, most obviously in time efficiency and the ability to be location agnostic.^6^ Technologies, such as video chat and electronic record keeping, that enable effective virtual consultations largely lie in the Web 2.0 space, and the connectivity that enables such interactions and working modalities can be adapted and extended to realms ranging from a family doctor consultation to that of remotely enabled surgery. The effectiveness of telehealth in its most rudimentary form can be comparable or superior to that of conventional physical clinic delivery, but is dependent on the disease indication and specific type of patient encounter, and may not be suitable for all patients. It may also exacerbate health inequalities because of the ‘Digital Divide’ between better educated and more affluent population groups compared to less well-educated and poorer groups.^7^

Developments in technology have paved the way for the augmentation of healthcare services delivery and digital therapeutics for conditions of high unmet need through virtual, augmented and mixed reality hardware, key enablers of a healthcare metaverse. An instance where this may be the case is in the clinical assessment of patients with musculoskeletal conditions and movement disorders through, for example, a virtual hologram that would allow for higher fidelity clinical interpretation and analysis of patients not located in the same physical space.^8^

The healthcare professional and patient interface may benefit from technologies such as the mixed reality headset, Microsoft HoloLens, which has been widely deployed in healthcare settings to help with medical education and training. Mixed reality headsets have been deployed in the context of guided surgery in the operating room or ward rounds where patient vitals and investigations can be projected onto the user’s visual landscape while at the patient’s bedside, reducing the ward round time by around 43 min or one-third when compared to the total time without such augmentation.^9^ The number of staff needing to be present on the ward round can also be cut down through telecommunication supported through such devices. Virtual reality, a fully immersive virtual experience, has been trialled effectively as a digital therapeutic for pain management and mental health.^10^

The metaverse will not, however, be a solution for all ailments or a solution for a healthcare system that has fundamental structural deficits and is under extreme pressures. There are significant financial and educational bottlenecks with the deployment and maturation of such technologies. Unintended consequences of social media and increased screen time could, for example, exacerbate mental health issues in adolescents. Rates of short-sightedness are rapidly rising and nearly 5 billion people will be myopic in 2050, but counterintuitively the problem could equally be the solution – social media could improve education for families and in turn modulate child behaviour reducing the risk factors for myopia progression, such as reducing screen time and increasing physical time outdoors.^11^

Legacy technology interoperability, the digital divide and technological adoption challenges for the elderly and disadvantaged are important factors to consider, as there is increasing concern that national and international health inequalities and inequities will be exacerbated by the technological arms race driven by intensifying technological and geopolitical rivalry.^12^ Digital therapeutics have already shown how the increasing widespread ubiquity of digital devices and user familiarity with technology can produce new solutions to the benefit of patients and healthcare systems. An example is the Food and Drug Administration-approved video game, EndeavorRx, to treat attention deficit hyperactivity disorder.^13^ The metaverse, however, promises much more for health. Our young people, and therefore health itself, will inevitably be shaped by the advent of the metaverse, enabling us to deliver on a better and optimistic future vision for health.^14,15^

## Declarations

### Guarantor

CSQ.

### ORCID iDs

Connor S Qiu https://orcid.org/0000-0002-7314-1504

Azeem Majeed https://orcid.org/0000-0002-2357-9858

### Provenance

Non commissioned; editorial review.
